# Transcranial alternating brain stimulation at alpha frequency reduces hemispatial neglect symptoms in stroke patients

**DOI:** 10.1016/j.ijchp.2022.100326

**Published:** 2022-08-05

**Authors:** Teresa Schuhmann, Felix Duecker, Marij Middag-van Spanje, Stefano Gallotto, Caroline van Heugten, Anne-Claire Schrijnemaekers, Robert van Oostenbrugge, Alexander T. Sack

**Affiliations:** a Section Brain Stimulation and Cognition, Department of Cognitive Neuroscience, Faculty of Psychology and Neuroscience, Maastricht University, Maastricht, the Netherlands; b Maastricht Brain Imaging Centre (MBIC), Maastricht University, Maastricht, the Netherlands; c InteraktContour, Nunspeet, the Netherlands; d EEG and Epilepsy Unit, University Hospitals and Faculty of Medicine of Geneva, University of Geneva, Geneva, Switzerland; e Limburg Brain Injury Center, the Netherlands; f Department of Neuropsychology & Psychopharmacology, Faculty of Psychology and Neuroscience (FPN), Maastricht University, the Netherlands; g School for Mental Health and Neuroscience, Department of Psychiatry & Neuropsychology, Faculty of Health, Medicine and Life Sciences (FHML), Maastricht University Medical Center, the Netherlands; h Adelante Rehabilitation Centre, Department of Brain Injury, Hoensbroek, the Netherlands; i Mondriaan Mental Health Centre, Department of Adult Psychiatry, Heerlen, the Netherlands; j Department of Neurology, School for Mental Health and Neuroscience, Maastricht University Medical Center, Maastricht, the Netherlands; k Centre for Integrative Neuroscience, Faculty of Psychology and Neuroscience, Faculty of Health, Medicine and Life Sciences, Maastricht University, Maastricht, the Netherlands

**Keywords:** Non-invasive brain stimulation, Transcranial alternating current stimulation, Neuropsychology, Visuospatial neglect

## Abstract

**Background/Objective:**

Non-invasive brain stimulation techniques such as transcranial alternating current stimulation (tACS) may help alleviate attention deficits in stroke patients with hemispatial neglect by modulating oscillatory brain activity. We applied high-definition (HD)-tACS at alpha frequency over the contralesional hemisphere to support unilateral oscillatory alpha activity and correct for the pathologically altered attention bias in neglect patients.

**Methods:**

We performed a within-subject, placebo-controlled study in which sixteen stroke patients with hemispatial neglect underwent 10 Hz (alpha) as well as sham (placebo) stimulation targeting the contralesional posterior parietal cortex. Attentional bias was measured with a computerized visual detection paradigm and two standard paper-and-pencil neglect tests.

**Results:**

We revealed a significant shift of attentional resources after alpha-HD-tACS, but not sham tACS, toward the ipsilateral and thus contralesional hemifield leading to a reduction in neglect symptoms, measured with a computerized visual detection paradigm and a widely used standard paper and pencil neglect tests.

**Conclusions:**

We showed a significant alpha-HD-tACS-induced shift of attentional resources toward the contralesional hemifield, thus leading to a reduction in neglect symptoms. Importantly, HD-tACS effects persisted after the stimulation itself had ended. This tACS protocol, based on intrinsic oscillatory processes, may be an effective and well-tolerated treatment option for neglect.

## Introduction

Each year more than 12 million people worldwide suffer from the devastating consequences of a new stroke, including severe cognitive deficits in attention and memory (Feigin et al., 2022). Among these cognitive deficits, visuospatial hemineglect is a common and disabling problem and is marked by the inability to attend to the contralesional side of space (Buxbaum et al., 2004; Osawa & Maeshima, 2021). These pronounced spatial attention deficits in hemineglect have a substantial negative impact on stroke patients’ everyday life and are a strong predictor of poor functional recovery (Di Monaco et al., 2011; Stone et al., 1992). Current rehabilitation options include a number of cognitive trainings, such as visual scanning training (VST), prism adaptation, or limb activation training. However, although the VST is generally advised as a preferred treatment option (ten Brink et al., 2016) and implemented in many rehabilitation centers, recent randomized controlled trials find only limited clinical benefits (Azouvi et al., 2017; Fasotti & van Kessel, 2013). To achieve higher clinical benefit, new treatment options have to be explored, possibly aiming at a neuromodulation of brain structures involved in visuospatial processing.

Fundamental neuroscientific research has started to unravel the functional organization and brain network communication underlying the control of spatial attention in the healthy brain (Morishima et al., 2009; Ruff et al., 2008; Sack et al., 2007), linking spatial attention bias to cortical excitability (Klimesch et al., 1998, 2007) and oscillatory activity in posterior parietal cortices (Fiebelkorn & Kastner, 2019). Modulating unilateral cortical excitability by noninvasive brain stimulation (NIBS) to create (or restore) an imbalance between competing hemispheres that suppress each other via interhemispheric inhibition, has shown to significantly affect spatial attention performance in a hemifield-specific way (Battelli et al., 2009; Bien et al., 2012; Dambeck et al., 2006; Hilgetag et al., 2001; Sack et al., 2002). Several small-scale clinical trials have tried to exploit this link between cortical excitability and attentional bias in patients with visuospatial neglect. In these studies, NIBS is applied to counteract the pathological attentional bias caused by the stroke through decreasing cortical excitability within the contralesional, i.e. unaffected, posterior parietal cortex, expecting to reduce its hyper-excitability and to thereby restore the interhemispheric balance. Unfortunately, although promising, reported clinical effects have remained rather small and heterogeneous (Koch et al., 2012; Lefaucheur et al., 2014, 2020; Longley et al., 2021).

NIBS protocols are not limited to modulating cortical excitability, but can also be tuned to influence oscillatory brain activity. Specifically, intrinsic brain oscillations can be amplified by alternating currents using transcranial alternating current stimulation (tACS) with the appropriate frequency, leading to entrainment and/or resonance effects (Lakatos et al., 2019). In the context of attention, oscillatory activity in the alpha range (8–12 Hz) over the posterior parietal cortex has been linked to attentional bias and attentional orienting (Thut et al., 2006; Worden et al., 2000). Mechanistically, it is often argued that alpha oscillations are crucial for gating information flow between different regions within a brain network by functional inhibition (Jensen & Mazaheri, 2010; Klimesch et al., 2007). Accordingly, shifting attention to the right hemifield is accompanied by alpha power decreases in the left hemisphere (release from inhibition) and alpha power increases in the right hemisphere (inhibiting the unattended left hemifield). Modulating this alpha power lateralization, instead of merely changing local cortical excitability, may therefore be a promising new and mechanistically different approach to correct for a pathological spatial attention bias after stroke using NIBS. Yet, until today, no study has tested this oscillation-based NIBS intervention in stroke patients suffering from visuospatial neglect to evaluate its feasibility and clinical efficacy.

Here, we present a proof-of-concept study for the use of high-definition transcranial alternating current stimulation (HD-tACS) in subacute stroke patients with visuospatial neglect aimed at reducing the visuospatial attention bias. To this end, we applied both sham and active HD-tACS at alpha frequency over the contralesional posterior parietal cortex in two different sessions. Based on the fundamental neuroscientific insights obtained in healthy volunteers outlined above, we expected an alpha-tACS-induced shift of attentional resources toward the ipsilateral and thus contralesional hemifield leading to a reduction in neglect symptoms measured with a novel computerized visual detection task and two widely used standard paper and pencil neglect tasks.

## Methods

### Study design

We performed a single center, within-subject, placebo-controlled study. Each patient underwent 10 Hz (alpha) as well as sham (placebo) stimulation in two separate HD-tACS sessions. The order of sessions was counterbalanced and the two sessions were performed on two different days with at least one-day inter-session interval. In both sessions, patients had to perform three different tasks, administered before (baseline), during, and immediately after HD-tACS. Patients gave written informed consent before participating in this experiment, in accordance with the 2008 Declaration of Helsinki and with the approval of the Medical Ethics Committee of the University Hospital Maastricht and Maastricht University (METC MUMC, registration number METC143030), The Netherlands.

### Participants

We recruited 17 subacute stroke patients from Adelante Rehabilitation center, Hoensbroek, The Netherlands in the period of October 2015 to April 2017. Patients with a recent clinically diagnosed first and/or recurrent stroke (ischemic or intracerebral haemorrhagic lesion) were considered eligible. Patients had to fulfill the inclusion criteria of having visuospatial neglect symptoms (either left- or right-sided neglect) based on clinical judgement and of having sufficient communication skills to understand the researcher's instructions. Patients were excluded if they had dementia and/or cochlear implants. Demographics (age, gender) and stroke-related characteristics (time since stroke, stroke type, stroke side) were collected from the patients’ medical records. Sixteen of the patients were right-handed, one patient was left-handed. Independence in activities of daily living (ADL) was assessed using the Barthel index (Collin et al., 1988) within 2 weeks after having been admitted to the rehabilitation center. Barthel scores ranged from 0 (completely dependent) up to 20 (completely independent).

### Transcranial alternating current stimulation

**Figure 1:**
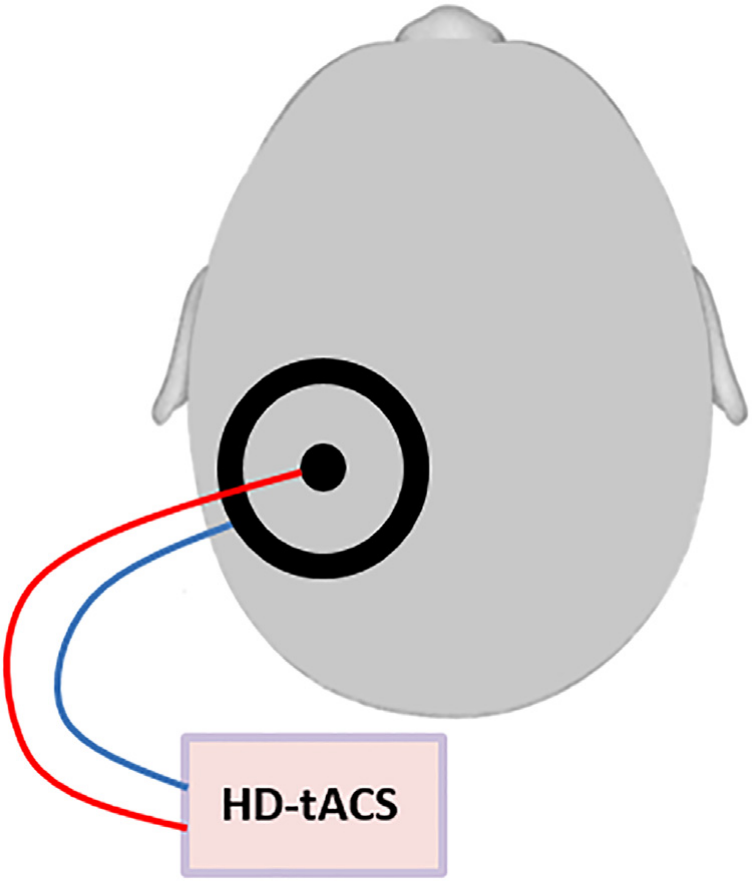
Schematic figure of the HD-tACS set-up: HD-tACS was performed using a small circular (diameter: 2.1 cm, thickness: 2 mm) and a large (outer diameter: 11 cm; inner diameter: 9 cm, thickness: 2 mm) rubber ring tACS electrode (NeuroConn, Ilmenau, Germany) that were both placed onto the contralesional posterior parietal cortex, with the small electrode positioned over P3 or P4 (based on the international 10–20 EEG system) and the large electrode centered on it.

HD-tACS was performed using a small circular (diameter: 2.1 cm, thickness: 2 mm) and a large (outer diameter: 11 cm; inner diameter: 9 cm, thickness: 2 mm) rubber ring tACS electrode (NeuroConn, Ilmenau, Germany) that were both placed onto the contralesional posterior parietal cortex, with the small electrode positioned over P3 or P4 (based on the international 10–20 EEG system) and the large electrode centered on it. This ring electrode montage enables a higher spatial focality as compared to standard rectangular electrodes (Datta et al., 2008) (Figure 1). Conductive gel (ten20 paste, Weaver and Company, Aurora, CO, USA) was applied between skin and electrodes to reduce the impedance to below 10 kΩ. Stimulation frequency and intensity were respectively set to 10 Hz and 1.5 mA peak to peak, phase offset was set to 0 and 100 cycles were used for ramping up. The control intervention consisted of sham stimulation and included ramping up and then immediately ramping down with each 100 cycles. This way, the patient feels the ramp up and ramp down events (which are the most noticeable in TES), but does not receive a significant dose of TES (Paulus et al., 2013). Unlike for TMS, this placebo/sham condition is indistinguishable from active HD-tACS for participants, ensuring successful blinding. Stimulation in both conditions lasted for maximally 30 min.

### Primary outcome: computerized visual detection task (CVDT)

The CVDT measures perceptual sensitivity and attentional selection in each hemifield separately, but also in the context of competition between visual stimuli in both hemifields. It is a simple and sensitive assessment of unilateral neglect and extinction (Bien et al., 2012; Duecker et al., 2017; Schuhmann et al., 2019). During the task, patients were seated in front of a computer screen at a distance of 57 cm. They were asked to fixate on the center of the screen, marked with a bull's-eye. Gabor patches (spatial frequency = 1.5 cycles per degree, envelope standard deviation = 0.75°, random orientation) were presented to the left, right and bilateral sides of the screen at 14° eccentricity. Stimuli were shown for 100 ms and stimulus size was 10° Patients had to verbally indicate whether they saw the stimulus appearing on the left, right or both sides of the fixation bull's-eye. For each trial, the stimulus position, contrast level, and response were recorded.

For each of the three locations (left, right, bilateral) independently, the contrast of the stimuli was adaptively changed on a trial-by-trial basis using the QUEST staircase algorithm (Watson & Pelli, 1983), as implemented in the Psychophysics Toolbox extension (Brainard, 1997) for MATLAB (The MathWorks, Inc., Natick, MA). We supplied the following parameters: prior mean was based on a short calibration procedure (see below), prior standard deviation = 1, beta = 3.5, gamma = 0.01, delta = 0.01, and aim performance = 0.5 (50% detection rate). The next contrast value was requested with QuestQuantile, and we obtained final detection threshold estimates with QuestMean.

Participants initially performed a short calibration procedure to obtain a first estimate of the individual detection threshold, which was used as a prior for the Bayesian staircase procedure. During this calibration, bilateral stimuli were presented on the screen, matching the positions used during the experimental task, and participants adjusted the contrast level of the stimuli until they could barely see them. At the beginning of the experimental task, two warm-up trials with high-contrast stimuli were included for each condition (left, right, and bilateral) that were easy to detect and not part of the staircase procedure. Then, participants completed three randomly interleaved staircases (left, right, bilateral) with 40 trials each. The overall duration of this task never exceeded 10 min.

Stimuli were presented on a Dell Latitude E6540 laptop. The video mode was 1920 × 1080 at 60 Hz, and background luminance was 105.55 cd/m2. The Presentation software package (NeuroBehavioural Systems, Albany, CA) was used to control stimulus presentation and recording of behavioral responses, interfacing with MATLAB for running QUEST functions.

### Secondary outcomes

We administered two neuropsychological paper-and-pencil tasks to assess the presence and severity of visuospatial neglect. The Bell's Task (BT) is a cancelation task which directly reflects the basic direction-specific deficit in visual searching (exploratory deficit) that is so characteristic of neglect patients’ clinical behavior (Ferber & Karnath, 2001). The test consists of an A4 sheet of paper with 315 black objects printed on it. Of the 315 objects, 35 are target items (bells) and the other 280 are distractor objects (houses, horses etc.). Although the objects appear to be presented in random order, they are distributed equally into 7 columns across the A4 sheet with 5 targets and 40 distractors per column. Patients were seated such that the center of the sheet was aligned to their midsagittal plane and instructed to circle all target items as quickly as possible. The total number of omitted targets was recorded, ranging from 0 to 35. The spatial distribution of the omitted targets determines the direction and severity of the visual neglect.

We also used the Line Bisection Task (LBT), which is a quick quantitative assessment of the presence and severity of unilateral spatial neglect (Schenkenberg et al., 1980). Line bisection necessitates correct perception of the size of a single stimulus, and a displacement of the bisection mark towards the ipsilesional side is interpreted as a symptom of neglect (Ferber & Karnath, 2001). The LBT requires patients to place a mark through the center of a series of 12 horizontal lines on a page placed in front of them. The test was scored by measuring the deviation in millimeters of the patient's bisection mark from the true center of the line. Deviations were scored positive for marks placed on the ipsilesional side of the center of the line and scored negative for marks placed on the contralesional side of the line-center (potential score range: −590 to +590 mm). Trials with omitted lines were scored as if patients put the mark all the way to the right or left side (in case of right or left hemisphere damage, respectively).

### Data analysis

Detection performance of the CVDT was tested in three conditions (ipsilesional stimulus, contralesional stimulus, bilateral stimulus), with the unilateral conditions directly relating to neglect symptoms, and the bilateral condition relating to extinction symptoms. Task performance was initially defined as detection thresholds for the three stimulus conditions. However, detection thresholds could not be used in some patients because they had so severe deficits that parameters were outside the test range and thus unreliable. The number of correct hits could be used as an alternative but this ignores the fact that contrast levels varied on a trial by trial basis, thus failing to take task difficulty into account. Hits were therefore weighted by the contrast level, according to the following formula: *x* = log10(max_contrast) / log10(trial_contrast). This measure accounts for the logarithmic nature of contrast detection, and makes trials count more when the contrast was low. This results in a potential scoring range of 0 to 76.49 weighted hits per condition. To illustrate, trials detected at maximum contrast received a score of 1, whereas trials with a relatively low contrast level of 10% received a score of 2. Performance in visual search as measured by the BT was tested in two conditions (contralesional side, ipsilesional side). To derive performance in the contralesional side, we calculated the average of missed targets in the three far-most contralesional columns, and to derive performance in the ipsilesional side, we calculated the average of missed targets in the three far-most ipsilesional columns.

Performance of the LBT was defined as the deviation of the patient's bisection mark from the true center of the line. The relative deviation was used to analyze the LBT data and was derived by means of the formula: *x* = deviation score / true half line length * 100. Relative deviation scores were then averaged across all 12 lines.

To quantify the patients’ spatial attention deficits, we analysed the baseline measurements (before stimulation) of both sessions (active and sham HD-tACS sessions averaged) per task. In the results we report our findings per task; always first showing the patients’ spatial attention deficits (sensitivity of the task), followed by the inference analyses performed using IBM SPSS Statistics Version 25. For all repeated-measures ANOVAs, we reported the multivariate test statistics (Pillai's trace). Follow-up analyses were conducted with paired t-tests. Significance was determined at *p*<.05.

## Results

We recruited seventeen subacute stroke patients from Adelante Rehabilitation center. One patient decided to stop participating after one session and was not included in the analyses. The final study sample comprised of sixteen patients, aged 37–76 years (*M* = 57.8 years, SD=9.7). Time since stroke ranged from 39 to 127 days (*M* = 87.4 days, SD=24.6). Patient characteristics are shown in Table 1. All patients included in the analysis were right handed.

**Table 1:** Patient characteristics (*n* = 16).

Characteristics	Outcome
Gender: males, *n* (%)	12 (75.0)
Age in years, mean ± SD (range)	57.8 ± 9.7 (37.1–76.1)
Time since stroke in days^a^, mean ± SD (range)	87.4 ± 24.6 (39.0–127.0)
Stroke type: *n* (%)	
Ischemic	10 (62.5)
Haemorrhagic	6 (37.5)
Stroke side: right, *n* (%)	15 (93.8)
Barthel index^b^, mean ± SD (range)	8.3 ± 7.1 (1.0–20.0)

a Time between stroke and baseline measurement of first session.

b Scores 1–20, higher score means higher degree of independence.

### Computerized visual detection task

Out of the sixteen patients, one patient was not able to perform the CVDT, and two patients displayed very high variability and were identified as statistical outliers (>3.0*IQR from Q1 and Q3). We here report the result of thirteen patients. All analyses of the CVDT were conducted on weighted hits. A repeated measures ANOVA of the baseline measurements averaged over both sessions, with Spatial Location (contralesional, bilateral, ipsilesional) as within-participant factor showed a significant effect of Spatial Location (F(2,11)=54.049, *p*<.00001, η_p_^2^ =0.908). Follow-up analyses showed a significant difference between contralesional and ipsilesional stimuli (t(12)=8.289, *p*<.00001) and between bilateral and ipsilesional stimuli (t(12)=7.115, *p*<.0001), demonstrating the strong attention deficits of the neglect patients in detecting stimuli in the contralesional hemifield. There was no significant difference between contralesional and bilateral stimuli (t(12)=0.176, *p*=.864), indicating that performance in both conditions was equally impaired.

**Figure 2:**
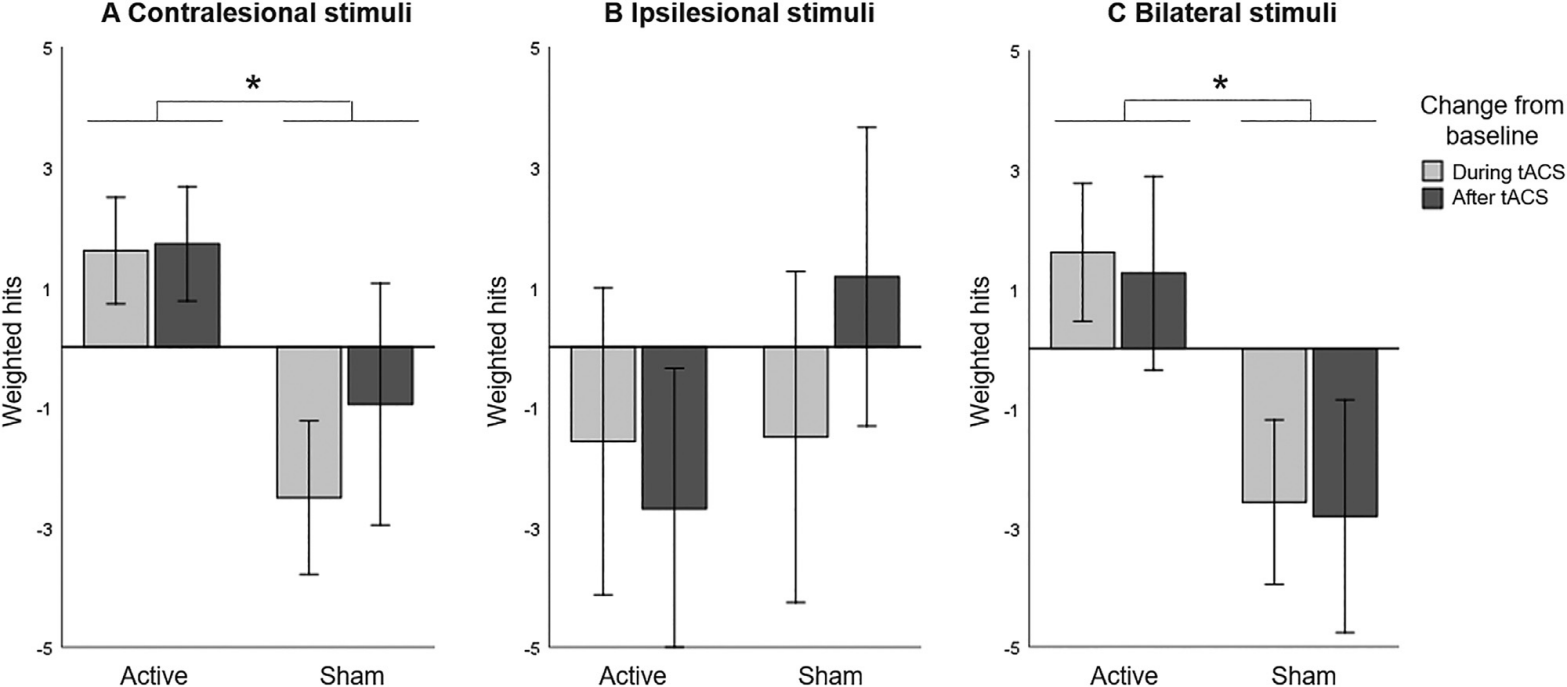
Computerized Visual Detection Task: Baseline corrected weighted hits (A) in contralesional hemifield, (B) in ipsilesional hemifield, and (C) when stimuli compete in both hemifields, for active and sham HD-tACS. A positive value indicates an improvement in detection performance over time (from baseline). A negative value indicates decreased performance compared to baseline, presumably due to increasing fatigue. Error bars depict one standard error. Asterisks (*) depict significant difference (*p*<.05).

The CVDT data was then split up to test the three hypotheses. First, we assessed the effect of unilateral HD-tACS stimulation over the contralesional parietal cortex on performance in the contralesional hemifield and expected an improvement in visual detection in active compared to sham tACS. Including only trials with stimuli in the contralesional hemifield, we performed a repeated measures ANOVA with HD-tACS (active, sham) and Time (baseline, during stimulation, after stimulation) as within-participant factors revealed no main effects of HD-tACS (F(1,12)=1.724, *p*=.214, η_p_^2^ =0.126) or Time (F(2,11)=0.729, *p*=.504, η_p_^2^ =0.117). However, the interaction between HD-tACS and Time (F(2,11)=8.895, *p*=.005, η_p_^2^ =0.618) was significant, indicating that the difference between detection performance before, during, and after stimulation was significantly different between the active alpha HD-tACS and the sham alpha HD-tACS stimulation conditions (Figure 2A). Regarding differences in performance between active and sham HD-tACS sessions, performance was equal at baseline (t(12)=0.975, *p*=.349), but during stimulation performance was significantly improved in the active compared to the sham session (t(12)=4.472, *p*=.001). This improvement was not significant after stimulation (t(12)=1.566, *p*=.143).

We then assessed the effect of unilateral HD-tACS stimulation on performance in the ipsilesional hemifield and expected no (or a negative) effect in visual detection performance in active compared to sham tACS. Including only trials with stimuli in the ipsilesional hemifield, A repeated measures ANOVA with HD-tACS (active, sham) and Time (baseline, during stimulation, after stimulation) as within-participant factors showed no main effect of HD-tACS (F(1,12)=0.901, *p*=.361, η_p_^2^ =0.070), or Time (F(2,11)=0.409, *p*=.674, η_p_^2^ =0.069), or interaction between these factors (F(2,11)=1.084, *p*=.372, η_p_^2^ =0.165) on the performance on the ipsilateral hemifield (Figure 2B).

Lastly, we assessed the effect of unilateral HD-tACS on performance when visual stimuli compete during bilateral presentation and expected an improvement in visual detection performance in active compared to sham sessions. Including only trials with bilateral stimuli, a repeated measures ANOVA analysing the performance on the bilateral trials, with HD-tACS (active, sham) and Time (baseline, during stimulation, after stimulation) as within-participant factors again revealed no main effect of Time (F(2,11)=0.106, *p*=.901, η_p_^2^ =0.019), but a significant main effect of HD-tACS (F(1,12)=5.179, *p*=.042, η_p_^2^ =0.301) and a significant interaction between HD-tACS and Time (F(2,11)=24.895, *p*<.0001, η_p_^2^ =0.819). Follow-up analyses revealed no difference at baseline between the two stimulations (t(12)=0.281, *p*=.783), but during the stimulation itself, performance was significantly improved in the active compared to the sham session (t(12)=3.209, *p*=.008) and this difference was still present after stimulation (t(12)=3.325, *p*=.006). Thus, HD-tACS affected performance during bilateral presentation of stimuli during and after the stimulation (Figure 2C).

### Bell's task

One patient was identified as statistical outlier (>3.0*IQR from Q1 and Q3), thus the data presented here includes 15 patients. To quantify the patients’ spatial attention deficits on the BT a paired-samples *t*-test on baseline measurements averaged over both sessions revealed a significantly higher average of omitted targets in the contralesional side compared to the ipsilesional side (t(14)=2.870, *p*=.012).

**Figure 3:**
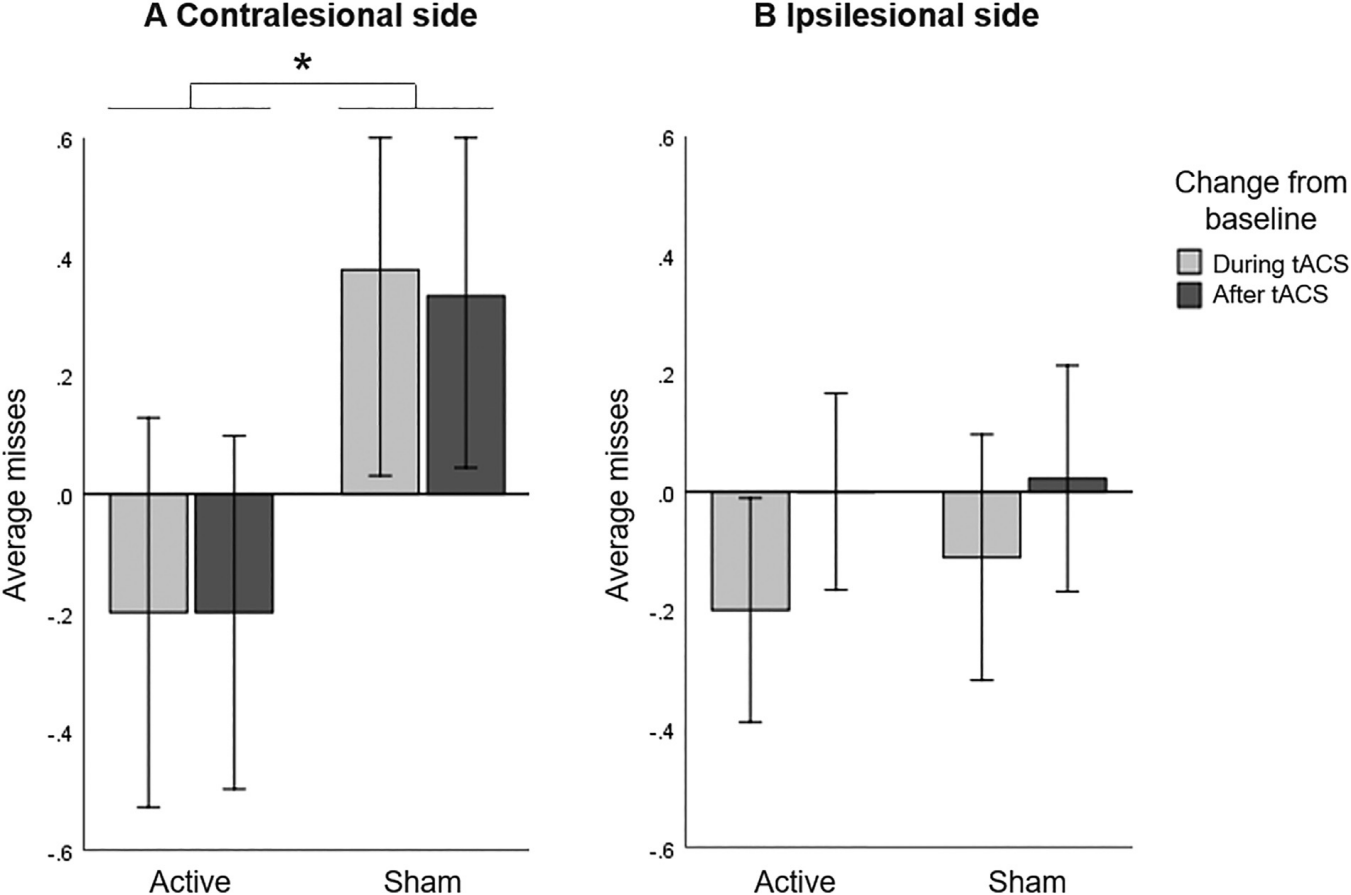
Bell's Task: Baseline corrected average misses (A) in contralesional side and (B) in ipsilesional side, for active and sham HD-tACS. A negative value indicates an improvement in performance in visual search over time (from baseline). A positive value indicates decreased performance compared to baseline, presumably due to increasing fatigue. Error bars depict one standard error. Asterisks (*) depict significant differences (*p*<.05).

A repeated measures ANOVA including only the number of missed bells in the contralesional side with HD-tACS (active, sham) and Time (baseline, during stimulation, after stimulation) as within-participant factors did not reveal a main effect of HD-tACS (F(1,14)=0.215, *p*=.650, η_p_^2^ =0.015) nor Time (F(2,13)=0.038, *p*=.963, η_p_^2^ =0.006), but it did reveal an interaction between HD-tACS and Time (F(2,13)=5.347, *p*=.020, η_p_^2^ =0.451). Since baseline differences between active and sham sessions (t(14)=2.578, *p*=.022) were found, we further explored the interaction by analysing changes from baseline. A repeated measures ANOVA on change scores, with HD-tACS (active, sham) and Time (during stimulation, after stimulation) as within-participant factors showed a main effect of HD-tACS (F(1,14)=7.261, *p*=.017, η_p_^2^ =0.342), but not Time (F(1,14)=0.021, *p*=.887, η_p_^2^ =0.001) nor an interaction between HD-tACS and Time (F(1,14)=0.015, *p*=.905). The average number of misses in the contralesional side was lower during and after stimulation in the active sessions as compared to the sham sessions (Figure 3A).

A repeated measures ANOVA including only the number of missed bells in the ipsilesional side with HD-tACS (active, sham) and Time (baseline, during stimulation, after stimulation) as within-participant factors revealed no main effects (HD-tACS (F(1,14)=0.009, *p*=.925, η_p_^2^ =0.001), Time (F(2,13)=2.836, *p*=.095, η_p_^2^ =0.304)) nor an interaction (F(2,13)=0.064, *p*=.939, η_p_^2^ =0.010). This implies that HD-tACS had no effect on the performance in the ipsilesional side (Figure 3B).

### Line bisection task

**Figure 4:**
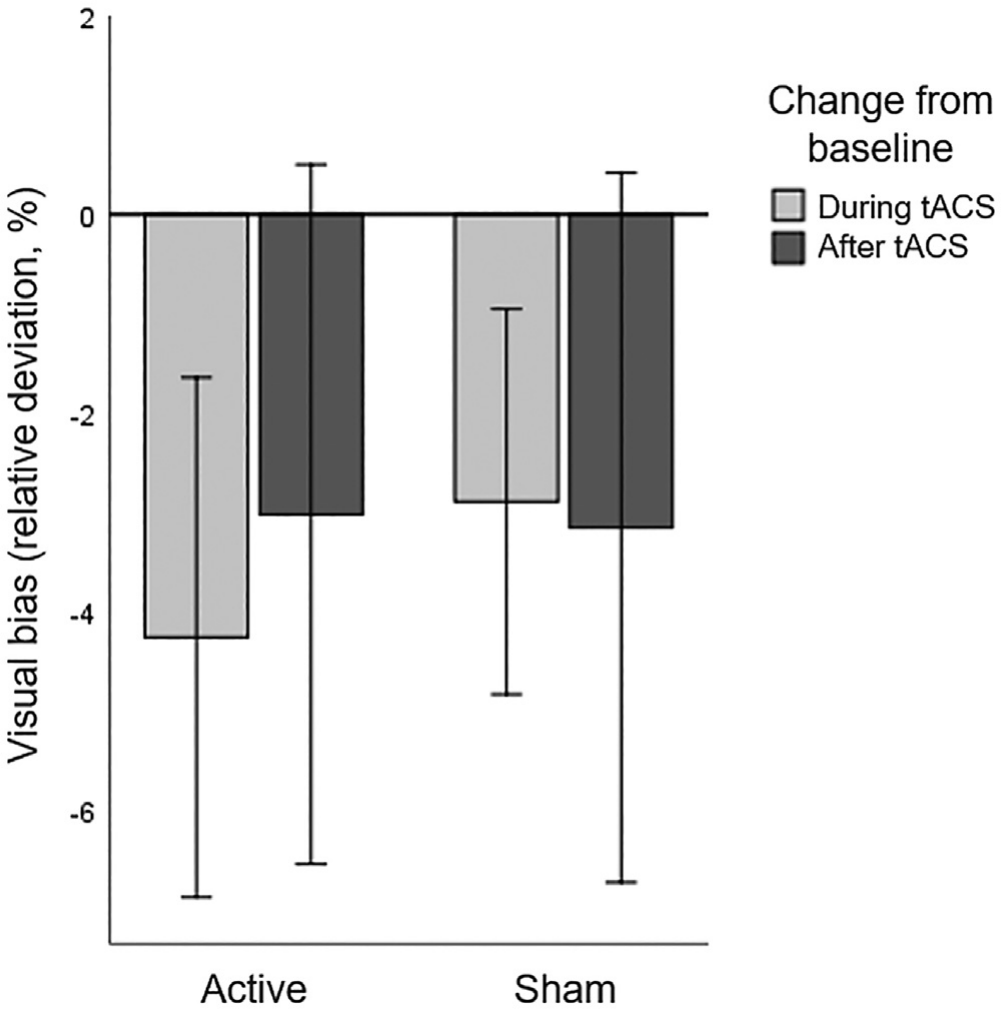
Line Bisection Task: Baseline corrected visual bias for active and sham HD-tACS. A negative value indicates that, compared to baseline, the bisection mark was placed less towards the ipsilesional side of space and more towards the contralesional (affected) side of space. Error bars depict one standard error.

No patients were identified as statistical outliers (>3.0*IQR from Q1 and Q3), and the data reported below is based on sixteen patients. Baseline performance on the LBT averaged over both sessions revealed a displacement of the bisection mark to the ipsilesional side, compared to 0 (t(15)=3.610, *p*=.003). A repeated measures ANOVA with HD-tACS (active, sham) and Time (baseline, during stimulation, after stimulation) as within-participant factors revealed no main effects of HD-tACS (F(1,15)=0.055, *p*=.818, η_p_^2^ =0.004) nor Time (F(2,14)=2.170, *p*=.151, η_p_^2^ =0.237) nor an interaction between HD-tACS and Time (F(2,14)=0.254, *p*=.779, η_p_^2^ =0.035) (Figure 4). This implies that tACS had no effect on the visual bias in the LBT.

## Discussion

This study aimed to alleviate attention deficits in hemineglect patients by using noninvasive transcranial brain stimulation to target functionally relevant oscillatory activity as a critical mechanism of attentional control. To this end, we applied high-definition transcranial alternating current stimulation (HD-tACS) at alpha frequency to the contralesional posterior parietal cortex of 17 hemineglect patients to modulate alpha power lateralization and to consequently correct their pathologically altered spatial attention bias. Compared to sham stimulation, patients significantly improved in allocating their attentional resources toward the contralesional hemifield leading to a reduction in neglect symptoms measured with a novel computerized visual detection paradigm (CVDT) and a widely used standard paper and pencil neglect task (Bell's Task, BT), but not on the Line Bisection Task (LBT). This effect could be seen in the unilateral/contralesional as well as the bilateral condition (measured with the CVDT), where performance depends on the contralesional and ipsilesional hemifield. Interestingly, the effects in the bilateral condition of the CVDT as well as the amount of misses in the BT in the contralesional side outlasted the stimulation time, meaning that the effect of the brain stimulation was still visible after stimulation. These results are the first proof-of-concept demonstration that this oscillatory-based transcranial stimulation approach is feasible, tolerable, and potentially clinically effective in treating hemineglect after stroke.

Our HD-tACS approach continues a recent trend towards directly targeting the biological basis for stroke-related impairments by non-invasive brain stimulation (NIBS). Previous studies aiming to reduce local cortical excitability in the contralesional hemisphere of patients with visuospatial neglect have produced some promising results, but the overall small and heterogeneous clinical effects at present only warrant a level-C recommendation according to the most recent European guidelines. Our hope is that improvements of efficacy can be made by tuning the brain stimulation protocol to the fundamental properties of network communication supported by oscillatory activity within and between functional brain networks.

This is exactly the mechanism based on which the here presented novel oscillation-based NIBS approach was developed. Instead of changing local cortical excitability, we aimed to entrain alpha oscillatory activity in the posterior parietal cortices to gate top-down selective information flow by functional inhibition (Jensen & Mazaheri, 2010; Klimesch et al., 2007). The oscillatory alpha band has been shown to be causally linked to such inhibitory gating with shifting attention to the right hemifield being accompanied by alpha power decreases in the left hemisphere (release from inhibition) and alpha power increases in the right hemisphere (inhibiting the unattended left hemifield).

We here show that modulating this alpha power lateralization with HD-tACS in neglect patients holds the potential to correct for a pathological spatial attention bias after stroke. Importantly, while classical excitability-based brain stimulation protocols often also achieve ipsilateral attention improvement but at the costs of contralateral attention impairments (shifting the balance towards the neglect side of space), our approach of enhancing alpha oscillatory activity in the left hemisphere did not negatively affect performance in the contralateral, i.e. ipsilesional hemifield as a consequence of the revealed significant performance improvement in the contralesional (neglected) hemifield. From a clinical standpoint, this novel brain stimulation approaches may therefore be more beneficial and desirable as compared to the current standard of decreasing unilateral excitability levels.

Interestingly, alpha-HD-tACS differentially affected the different paradigms used to assess attentional performances in our patient sample. The absence of effects on the LBT is somewhat unexpected, and likely due to compensatory strategies patients learned during cognitive training. This compensatory effect in the LBT has been reported previously (Keller et al., 2005).

A second possible explanation is that a deviation in line bisection is not fundamentally related to spatial neglect, but may also arise from disturbances of other sensory and cognitive processes, such as hemianopia (Ferber & Karnath, 2001). Our tACS-therapy targets attentional processes in the brain and does not treat visual deficits. It may well be that some neglect patients in our sample also suffered from visual field deficits, since visual neglect and visual field deficits commonly co-occur after unilateral brain damage such as stroke (Halligan, 1999). In a study that compared the accuracy of the LBT and cancelation tests (including the BT) in detecting spatial neglect, cancelation tests proved to be far superior, suggesting they reflect spatial neglect symptomatology more distinctly (Ferber & Karnath, 2001). This demonstrates that carefully selected tasks are very relevant to reveal attention deficits. Even though the BT worked as intended in our current study, we believe adaptive testing as used here during our CVDT is very promising as it allows assessment across the entire spectrum of neglect severity (at least in the ideal case).

We were able to show immediate stimulation effects, but also effects outlasting the stimulation itself. This not only suggests that our approach does qualify for a clinical treatment protocol aimed at achieving longer lasting after-effects, but also indicates that the task-specific effects we find are not confounded by the stimulation itself. It should be noted that the current study only included 16 patients, and future studies with more patients are recommended. Future studies could also include electroencephalography (EEG), not only to measure and show potential changes in oscillations after stimulation, but also to individualize the stimulation parameters themselves. We were able to show in a healthy population group that stimulation at the individual frequency, compared to stimulation at flanker frequencies lead to larger alpha lateralization after stimulation (Kemmerer et al., 2022). The oscillatory-based approach described thus allows personalizing the treatment protocol by stimulating based on individual oscillatory frequency parameters (Zaehle et al., 2010) but it also allows extending to different frequency bands. In addition, HD-tACS has shown to be a very well tolerated, feasible, low cost and portable technique, which therefore lends itself perfectly to be amended by cognitive training and even used in a home-setting (at-home use with remote supervision). Based on this proof-of-concept, a larger randomized controlled clinical trial is needed to evaluate the clinical efficacy of many repeated treatment sessions over the course of rehabilitation to hopefully induce long-lasting changes, which has already been demonstrated in psychiatric disorders, such as depression.

## Conclusions

Administering HD-tACS at alpha frequency over the contralesional hemisphere improves spatial attention deficits in subacute stroke patients. Oscillatory-based tACS might be a promising therapeutic tool in patients with attentional deficits.

## Funding

This research did not receive any specific grant from funding agencies in the public, commercial, or not-for-profit sectors.

## Declaration of Competing Interest

None.
